# Evaluating the patient sociodemographic factors affecting dental students' clinical communication skills using a three‐perspective approach

**DOI:** 10.1002/cre2.897

**Published:** 2024-06-16

**Authors:** Siti Mariam Ab Ghani, Puteri Nurul Adila Mohd Khairuddin, Budi Aslinie Md Sabri, Dieter Schonwetter, Tong Wah Lim

**Affiliations:** ^1^ Centre of Restorative Dentistry Studies, Faculty of Dentistry Universiti Teknologi MARA, Sungai Buloh Campus Sungai Buloh Selangor Malaysia; ^2^ Center of Population Oral Health and Clinical Prevention Studies Faculty of Dentistry, Universiti Teknologi MARA Sungai Buloh Campus Sungai Buloh Selangor Malaysia; ^3^ Dr. Gerald Niznick College of Dentistry University of Manitoba Winnipeg Manitoba Canada; ^4^ Division of Restorative Dental Sciences Faculty of Dentistry, The University of Hong Kong Sai Ying Pun Hong Kong SAR

**Keywords:** demography, dentist–patient relations, interpersonal relations, patients

## Abstract

**Objective:**

This study aimed to assess undergraduate dental students' communication skills in relation to patient sociodemographic factors using a three‐perspective approach; the student, the patient, and the clinical instructor perspective.

**Materials and Methods:**

A cross‐sectional study was conducted using validated modified‐communication tools; Patient Communication Assessment Instruments (PCAI), Student Communication Assessment Instruments (SCAI), and Clinical Communication Assessment Instruments (CCAI). Moreover, 176 undergraduate clinical year students were recruited in this study whereby each student was assessed by a clinical instructor, a patient, and self‐evaluation.

**Results:**

The clinical communication skills domains were not significantly influenced by patient sociodemographic factors, including sex, educational background, and the number of visits (*p* > .05). However, this study revealed a statistically significant difference in the domain of “caring and respectful” of the SCAI between the low‐ and middle‐income groups.

**Conclusions:**

Overall, most of the patient sociodemographic factors did not affect clinical communication skills. However, patient income groups played a significant role in one of the communication domains.

## 1 INTRODUCTION

Effective communication between patient and clinician is crucial, and it can be defined as the exchanging of verbal and nonverbal messages between two parties to share their information and emotions (Brooks & Heath, 1985). Effective therapeutic communication skills (Schönwetter, Wener, Mazurat, et al., 2012; Schönwetter, Wener, Mazurat, 2012) will enhance the clinicians' diagnostic efficiency, ensure that a shared decision‐making approach between clinician and patient is applied (Lim et al., 2018; Mustaza et al., 2016), and is an integral part of the competency‐based training for all health professionals (Tavakoly Sany et al., 2020). It improves patient satisfaction and facilitates patient adherence to the clinician's recommendation. Moreover, effective communication will reduce patient anxiety, complaint incidences, and medical errors (van der Molen et al., 2004). The dental students are advised to connect and communicate with their patients, establishing a good patient–clinician relationship from the very first visit. In addition, the communication skills training in dental school is also highly valued by the students (Hannah et al., 2004). Finally, the Healthy People 2010 national health survey and the most recent Healthy People 2020 have stated the importance of good communication between healthcare providers and patients to eradicate oral health disparities (Laurence et al., 2012).

Various patient factors influence the delivery of effective communication, including age, sex, educational background, socioeconomic status, and past treatment experience. A systematic review concluded that older respondents were significantly more satisfied than younger participants in 70% of the included studies (Crow et al., 2002). Although older patients tended to be more satisfied with the dentist's attentiveness, they might not be as content with the extent of communication and care (Ali, 2016; Stege et al., 1986). In terms of sex differences, the findings have been varied (Arnbjerg et al., 1992; Gopalakrishna & Munnaleneni, 1993; John et al., 2011; Newsome & Wright, 1999; Skaret et al., 2005; Woods & Heidari, 2003). Satisfaction with dental care among genders may be influenced by pain management during treatment (Skaret et al., 2005) and the frequency of dentist visits (Gopalakrishna & Munnaleneni, 1993).

Socioeconomic status was strongly associated with the levels of education, income, and occupation (Gürdal et al., 2000). Educational level played a significant role in communication satisfaction among patients, with low satisfaction reported among highly educated patients due to their higher expectations of dental services (Gürdal et al., 2000; John et al., 2011; White et al., 2001). On the contrary, Theaker et al. (2000) reported that university‐educated patients demonstrated high satisfaction scores because they were greeted in a friendly manner and felt heard when asking questions. This finding aligns with other studies reporting that patients with high socioeconomic status were more satisfied with dental services because they appreciated the interpersonal skills of clinicians and were better able to understand and analyze the information provided to them compared to patients with lower socioeconomic status (Theaker et al., 2000; Willems et al., 2005).

Positive and negative associations have been observed between satisfaction levels and past treatment experiences (Gürdal et al., 2000; Newsome & Wright, 1999; Schönwetter, Wener, Mazurat, et al., 2012; Sondell et al., 2002; White et al., 2001). Patients and dental student clinicians were able to develop closer relationships after longer appointment durations or an increased number of dental treatments for patients (Sondell et al., 2002; White et al., 2001). Patients tended to evaluate their dental student clinician's communication skills more favorably during their first visit, as only simple and nonaggressive procedures were performed compared to following visits (Schönwetter, Wener, Mazurat, et al., 2012). Conversely, students' confidence levels could also be negatively impacted by increased treatment durations, which in turn affected their communication with patients (Gürdal et al., 2000).

It is evident that dental student clinicians' communication skills are essential for enhancing patient satisfaction. Some of the findings from this cross‐sectional study, including the sex of students, years of clinical experience, and clinical setting, have been previously published (Ab Ghani et al., 2023). Numerous patient factors influence the delivery of information and the effectiveness of communication between clinicians and patients. However, the nature of this relationship remains unclear, and limited data is available from the perspectives of patients, students, and clinical instructors. Therefore, this study aimed to assess undergraduate dental students' communication skills in relation to patient sociodemographic factors using a three‐perspective approach: self‐evaluation, the patient, and the clinical instructor perspective. The null hypothesis was that patient sociodemographic factors do not affect students' clinical communication skills.

## 2 MATERIALS AND METHODS

### 2.1 Study design and participants

**Figure 1:**
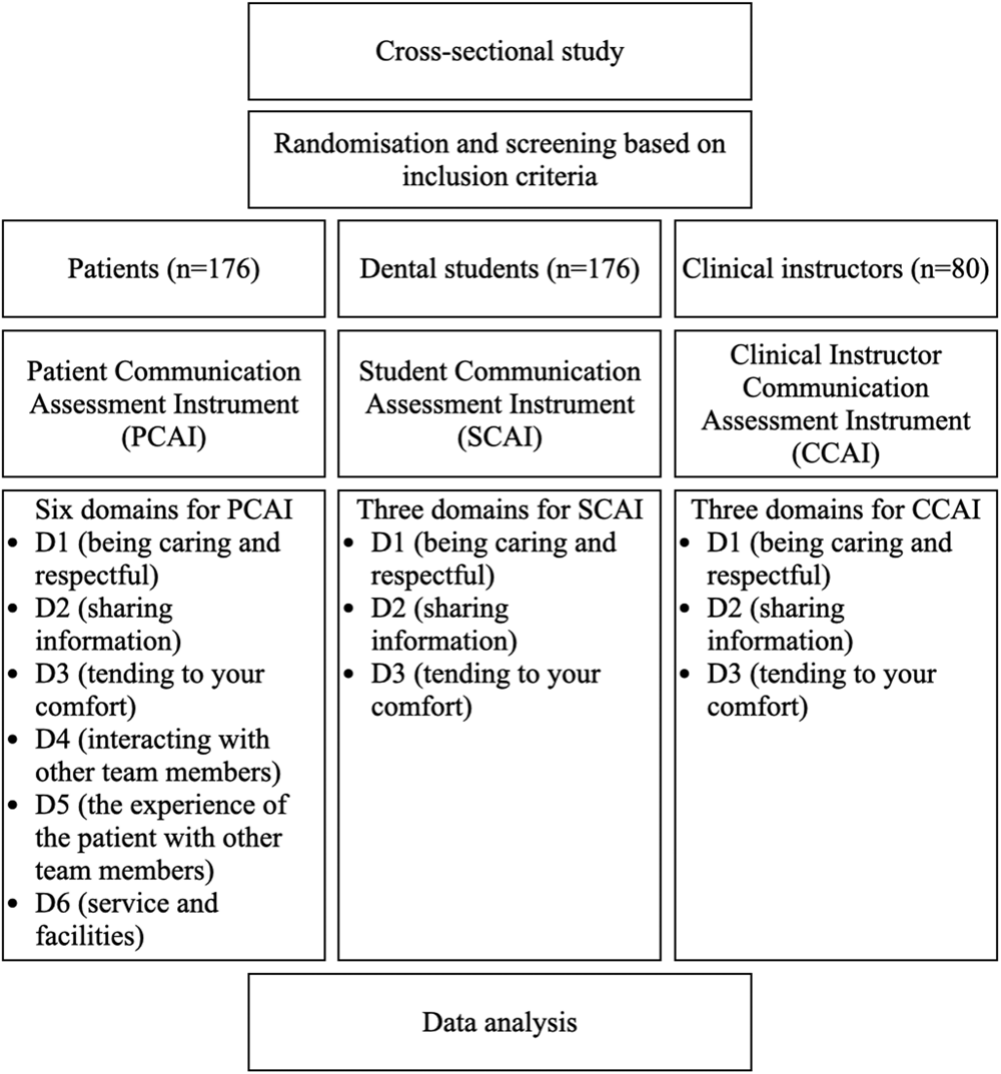
Flow diagram for this cross‐sectional study.

Ethics approval was obtained from the Research Ethics Committee of the university (Ref. No: 600‐IRMI‐5/1/6 REC/370/16). This cross‐sectional study was conducted using a validated questionnaire (Schönwetter, Wener, Mazurat, 2012) involving three groups of respondents: dental students, patients, and clinical instructors, as reported by Ab Ghani et al. (2023) A total of 176 student respondents consisting of clinical year students and their patients were recruited (Figure 1). The patients were randomly selected to avoid bias using a simple random sampling technique. All the students' names, together with their treated patients for each clinical session, were obtained from the student clinics and compiled, assigned a number, and randomly selected by the researcher (P. N. A. M. K.) not involved in the assessment using a random number generator. Ten pairs of participants (student and patient) who complied with the following inclusion criteria for each clinical session were recruited into the study until the projected sample size was achieved. The inclusion criteria were a patient aged 18 years and above with a sufficient understanding of the Malay language or English. Clinical instructors only randomly assessed five dental student clinicians assigned by the same researcher (P. N. A. M. K.) using the same generator at each clinical session to focus on their evaluation which produced a sum of 80 samples. All the participants were asked to sign a written consent form before completing the questionnaires. In addition, participants were advised to provide honest answers to improve student learning, ensuring that their responses would be kept anonymous and would not affect the grades of the dental student clinicians.

### 2.2 Study tools

The Student Communication Assessment Instrument (SCAI), Patient Communication Assessment Instrument (PCAI) (Schönwetter, Wener, Mazurat, et al., 2012; Schönwetter, Wener, Mazurat, 2012), and Clinical Instructor Communication Assessment Instrument (CCAI) (Ab Ghani et al., 2023) were used in this study (Figure 1). All six domains for these three instruments, namely: being caring and respectful (D1—16 items), sharing information (D2—12 items), tending to your comfort (D3—eight items), interacting with other team members (D4—four items), the experience of the patient with other team members (D5—11 items), and service and facilities (D6—five items). Each item utilized the Likert‐type response statements (“1 = poor,” “2 = satisfactory,” “3 = good,” “4 = very good,” and “5 = excellent”). The PCAI was translated into a Malay language using a forward and backward translation approach, and content validation using a scale‐level index (S‐CVI) was performed. The S‐CVI is defined as “the proportion of items given a rating of quite or very relevant by both raters involved” (Waltz et al., 2016), and 0.9 was scored for the present study. All the clinical instructors were calibrated during the pilot study to ensure standardization before the actual data collection.

### 2.3 Data collection

The present study was conducted at student dental clinics. All the questionnaires have been deidentified and kept anonymous by using an assigned reference number for the participants by the researcher (P. N. A. M. K.) not involved in the assessment. Patients were informed to complete the questionnaire (PCAI) at the end of the treatment session, away from the presence of the dental students, to encourage true scoring. Self‐evaluation using SCAI was also completed by the dental students concurrently to minimize bias. Clinical instructors evaluated the dental students using CCAI during the same clinical sessions. Patient sociodemographic data, including sex, age, educational background, income, and the number of visits, were recorded. The ages within the sample ranged from 18 years and above and were collected according to the United Nations Statistics Division's age classification (Appendix 1). Upon data analysis, participants were classified as adolescents (under 20 years old), adults (20–69 years old), and older adults (70 years old and above) based on the World Health Organization (http://www.who.int). Whereas the income groups: low‐, middle‐, and high‐income were categorized based on the Report of Household Income and Basic Amenities Survey 2016, Malaysians.

### 2.4 Statistical analysis

Data were entered and analyzed using the Statistical Package for Social Sciences (SPSS) software program version 24 (IBM SPSS Statistics for Windows, Version 24.0; IBM Corp). Descriptive statistics were employed to present the demographic data. Analysis of variance (ANOVA) or independent t‐test was performed to analyze the sociodemographic factors in each perspective, excluding the age of patients. The report of different age groups revealed a small sample size in some comparison cells. Consequently, descriptive statistics were utilized for this factor. A *p* value of <.05 was considered significant for the statistical tests performed.

## 3 RESULTS

A total of 432 questionnaires were collected, comprising 176 patient assessments (PCAI), 176 corresponding student self‐assessments (SCAI), and 80 clinical instructor assessments (CCAI). More than half of the patient respondents were females (*n* = 93, 52.8%). In addition, most of them were adults (*n* = 164, 93.2%), followed by nine (5.1%) who were adolescents and three (1.7%) who were older adults (Appendix 1). For the education group, 109 (61.9%) were highly educated, 60 (34.1%) were in secondary education, and seven (4%) were at the primary educational level. Moreover, 127 (72.2%) of the patient respondents were from the low household‐income group, 42 (23.9%), and seven (4.0%) were from the middle‐ and high‐income groups, respectively. In addition, 86.9% of the patients reported that they had three or more appointments with their students before the study, and the remainder (13.1%) had one to two appointments.

In general, all the domains in the PCAI for adolescents exhibited the highest scores ranging from 4.02 ± 0.78 to 4.27 ± 0.64 compared with the other age groups. In addition, the mean scores of all the domains in the PCAI and CCAI were the highest and the lowest, respectively, for all age groups. Table 1 summarizes the mean score of the communication domains in relation to patient gender and found that all the domains in the three instruments were not significantly related (*p* > .05) to sex. All the domains in the three instruments were not significantly related (*p* > .05) to the patient educational levels and the number of visits (Tables 2 and 3). Table 4 shows the distribution of communication domains in relation to the patient's median household income. There were significant differences (*p* < .05) found in D1, caring and respectful (*p* = .016) for SCAI. Post Hoc Tests revealed that a statistically significant difference (*p* = .012) was found between the low‐income group (3.73 ± 0.57) and the middle‐income group (4.03 ± 0.59) (Table 4).

**Table 1:** Comparison of communication domain scores in relation to the sex of patients.

Assessment	Domain	Male	Female	*t* Stats	*p* Value
		*n* = 83	*n* = 93		
		Mean (SD)	Mean (SD)		
SCAI	D1	3.84 (0.63)	3.78 (0.55)	0.419	0.518
	D2	3.85 (0.65)	3.75 (0.57)	1.251	0.265
	D3	3.84 (0.64)	3.84 (0.53)	0.001	0.971
PCAI	D1	4.11 (0.62)	4.18 (0.60)	0.492	0.484
	D2	4.01 (0.71)	4.11 (0.59)	1.093	0.297
	D3	4.09 (0.71)	4.18 (0.66)	0.750	0.388
	D4	3.98 (0.89)	4.03 (0.94)	0.147	0.702
	D5	4.06 (0.70)	3.99 (0.91)	0.273	0.602
	D6	4.06 (0.66)	3.95 (0.77)	1.014	0.315
CCAI	D1	3.57 (0.48)	3.57 (0.41)	0.003	0.959
	D2	3.49 (0.59)	3.48 (0.52)	0.002	0.962
	D3	3.53 (0.67)	3.45 (0.76)	0.308	0.581

Abbreviations: CCAI, Clinical Instructor Communication Assessment Instrument; D1, caring and respectful; D2, sharing information; D3, tending to comfort; D4, interacting with other dental team members; D5, experience with other dental team members; D6, service and facility; PCAI, Patient Communication Assessment Instrument; SCAI, Student Communication Assessment Instrument; SD, standard deviation.

*Significant at *p* < .05, independent *t* test.

**Table 2:** Comparison of communication domain scores in relation to the educational level of patients.

Assessment	Domain	Primary education	Secondary education	Higher education	*F* stats	*p* Value
		*n* = 7	*n* = 60	*n* = 109		
		Mean (SD)	Mean (SD)	Mean (SD)		
SCAI	D1	3.54 (0.58)	3.78 (0.61)	3.83 (0.58)	0.895	0.411
	D2	3.43 (0.53)	3.81 (0.61)	3.81 (0.61)	1.313	0.272
	D3	3.64 (0.57)	3.83 (0.61)	3.86 (0.57)	0.479	0.620
PCAI	D1	3.99 (0.71)	4.06 (0.59)	4.20 (0.61)	1.289	0.278
	D2	4.06 (0.62)	4.03 (0.61)	4.08 (0.68)	0.098	0.906
	D3	4.00 (0.65)	4.12 (0.63)	4.16 (0.72)	0.217	0.805
	D4	3.96 (0.80)	4.05 (0.85)	3.98 (0.97)	0.120	0.887
	D5	3.78 (0.75)	4.14 (0.59)	3.98 (0.93)	1.065	0.347
	D6	3.89 (0.84)	4.06 (0.65)	3.98 (0.76)	0.342	0.710
CCAI	D1	3.40 (0.34)	3.51 (0.41)	3.64 (0.47)	1.251	0.292
	D2	3.32 (0.51)	3.40 (0.53)	3.57 (0.57)	1.125	0.330
	D3	2.93 (0.98)	3.49 (0.55)	3.55 (0.79)	1.742	0.182

Abbreviations: CCAI, Clinical Instructor Communication Assessment Instrument; D1, caring and respectful; D2, sharing information; D3, tending to comfort; D4, interacting with other dental team members; D5, experience with other dental team members; D6, service and facility; PCAI, Patient Communication Assessment Instrument; SCAI, Student Communication Assessment Instrument; SD, standard deviation.

*Significant at *p* < .05, analysis of variance.

**Table 3:** Comparison of communication domain scores in relation to the number of visits of patients.

Assessment	Domain	1–2 appointments	3 or more appointments	*t* Stats	*p* Value
		*n* = 23	*n* = 153		
		Mean (SD)	Mean (SD)		
SCAI	D1	3.78 (0.73)	3.81 (0.57)	0.070	0.792
	D2	3.67 (0.76)	3.81 (0.59)	1.076	0.301
	D3	3.71 (0.71)	2.86 (0.56)	1.288	0.258
PCAI	D1	4.11 (0.67)	4.15 (0.60)	0.083	0.774
	D2	4.07 (0.65)	4.06 (0.65)	0.002	0.962
	D3	4.09 (0.84)	4.15 (0.66)	0.187	0.666
	D4	3.90 (1.08)	4.02 (0.90)	0.344	0.558
	D5	3.85 (1.06)	4.05 (0.78)	1.179	0.279
	D6	4.07 (0.53)	3.99 (0.75)	0.229	0.633
CCAI	D1	3.34 (0.34)	3.58 (0.44)	1.118	0.294
	D2	3.48 (0.58)	3.48 (0.55)	0.000	0.988
	D3	2.97 (1.28)	3.51 (0.67)	2.253	0.137

Abbreviations: CCAI, Clinical Instructor Communication Assessment Instrument; D1, caring and respectful; D2, sharing information; D3, tending to comfort; D4, interacting with other dental team members; D5, experience with other dental team members; D6, service and facility; PCAI, Patient Communication Assessment Instrument; SCAI, Student Communication Assessment Instrument; SD, standard deviation.

*Significant at *p* < .05, independent *t* test.

**Table 4:** Comparison of communication domain scores in relation to the income of patients.

Assessment	Domain	Low‐income	Middle‐income	High‐income	*F* stats	*p* Value	Post hoc Tukey adjustment (*p* value)
		*n* = 127	*n* = 42	*n* = 7			
		Mean (SD)	Mean (SD)	Mean (SD)			
SCAI	D1	3.73 (0.57)	4.03 (0.59)	3.86 (0.65)	4.247	*0.016	Low‐ × middle‐income (*0.012)
							Low‐ × high‐income (0.834)
							Middle‐ × high‐income (0.752)
	D2	3.74 (0.58)	3.98 (0.65)	3.70 (0.70)	2.721	0.069	
	D3	3.80 (0.58)	4.00 (0.58)	3.64 (0.63)	2.354	0.098	
PCAI	D1	4.10 (0.58)	4.31 (0.67)	4.03 (0.65)	2.140	0.121	
	D2	4.04 (0.60)	4.12 (0.79)	4.04 (0.71)	0.203	0.817	
	D3	4.11 (0.65)	4.27 (0.74)	3.91 (0.86)	1.214	0.299	
	D4	4.05 (0.83)	3.87 (1.18)	4.11 (0.73)	0.635	0.531	
	D5	4.06 (0.69)	3.95 (1.13)	3.90 (0.90)	0.365	0.695	
	D6	3.94 (0.73)	4.20 (0.68)	3.86 (0.75)	2.251	0.108	
CCAI	D1	3.59 (0.37)	3.46 (0.54)	3.88 (0.98)	1.309	0.276	
	D2	3.51 (0.51)	3.39 (0.67)	3.58 (0.58)	0.351	0.705	
	D3	3.50 (0.69)	3.41 (0.83)	3.67 (0.59)	0.193	0.825	

Abbreviations: CCAI, Clinical Instructor Communication Assessment Instrument; D1, caring and respectful; D2, sharing information; D3, tending to comfort; D4, interacting with other dental team members; D5, experience with other dental team members; D6, service and facility; PCAI, Patient Communication Assessment Instrument; SCAI, Student Communication Assessment Instrument; SD, standard deviation.

* Significant at *p* < .05, analysis of variance.

## 4 DISCUSSION

The present study investigated the patient sociodemographic factors affecting the communication skills between dental student clinicians and patients from three perspectives: patient, clinical instructor, and student self‐assessment. The domains in these three instruments were significantly affected by the income group, thus partially rejecting the null hypothesis. The assessments of communication skills in contemporary dental education are still in the early stage where there is no single approach that can be considered the gold standard. A 360° review approach is reported to be superior to other traditional forms of evaluation as this method is known to initiate a vast positive change and provides a more efficient, comprehensive, and accurate assessment of performance reviews (Ab Ghani et al., 2023; Mathew et al., 2015). Alternative approaches that could be considered include the Patient Assessment Questionnaire (Hurst et al., 2004), Dental Consultation Communication Checklist (Theaker et al., 2000), Objective Structured Clinical Examination (Baig et al., 2009), and Problem‐based Learning (Razzaq & Ahsin, 2011). Some studies employing this 360° approach may also include peer assessment; however, as the focus of this research pertains to communication during clinical practice and not throughout the entire education environment, the triad of patients, self, and clinical supervisor is considered sufficient.

In general, adolescents gave the highest scores in PCAI when compared to the other age groups. Possibly, the older patients have higher expectations from the students compared with the young patients, and the finding was supported by Ali (2016). However, this finding may be biased by the small sample size in the adolescent and older adult groups. Nevertheless, All communication domains within the SCAI and CCAI demonstrated consistent results across all age groups, indicating that patients and clinicians exhibited similar communication patterns regardless of age, as observed in both self‐evaluation and the clinical instructor's perspective.

The present study showed no statistical association between the sex of the patients and the communication skills of dental student clinicians. This finding is consistent with the study conducted by Arnbjerg et al. (1992) but in contrast to the others (Gopalakrishna & Munnaleneni, 1993; Newsome & Wright, 1999). In general, females bring more empathy, display kindness, and show greater attention to detail compared to males (Sondell et al., 2002). However, this study has shown that dental student clinicians can communicate equally well with both male and female patients. Possibly, the students were trained effectively during the communication skills module in dental school.

The educational background of the patients did not significantly affect the communication domains in PCAI, SCAI, and CCAI. This result may be explained by the good interpersonal skills of the dental student clinicians and the use of simple language as opposed to medical jargon in health communication. The finding is consistent with the previous studies, reporting that the relationship between education level and patient satisfaction was not statistically significant (John et al., 2011; Willems et al., 2005). However, patients with higher as compared to lower, academic qualifications may have higher expectations and more difficulty being satisfied (White et al., 2001).

In contrast to the findings of Schönwetter, Wener, Mazurat, et al. (2012), the present study reported that the domains in the three instruments were not significantly associated with the number of visits, possibly because the dental student clinicians and patients can communicate well with each other throughout the whole treatment process regardless of the number of patient visits. A good rapport between the patients and dental students may be developed after a long duration of prosthesis appointments, and high patient satisfaction can be achieved after more extensive dental treatments are performed (Sondell et al., 2002; White et al., 2001).

This study also revealed a statistically significant difference in the domain of “caring and respectful” of SCAI between the low‐income and the middle‐income group, where the dental student clinicians were found to be more caring toward the middle‐income group. Possibly, the students found that difficult to include them in discussion or to connect with them during the follow‐up review after the treatments compared with other groups. However, this finding may be confounded by other factors, including job background. The remaining communication domains for PCAI, SCAI, and CCAI did not show any statistically significant difference, which suggests that dental student clinicians do not discriminate against their patients' income levels when communicating to provide dental health services. Nonetheless, Willems et al. (2005) found that the patients with high socioeconomic status were able to understand and analyze the information given to them better when compared to the lower socioeconomic status group.

Limitations of this study include its cross‐sectional design, as questionnaires were administered after lengthy clinical appointments, potentially affecting participants' motivation to answer. Additionally, the lack of blinding among dental students in this study may introduce performance bias. Furthermore, the sample distribution was imbalanced particularly the age group classification, which could potentially lead to biased results. All the participants in this study took 5–15 min to complete answering the questionnaires. Longitudinal cohort studies with a better sample distribution are recommended to further analyze the results, particularly age groups.

## 5 CONCLUSIONS

The use of PCAI, SCAI, and CCAI together provided a complementary view of students' communication performance in all the assessed domains from three different perspectives. These three instruments have shown that dental student clinicians can communicate similarly and effectively with all patients regardless of their sociodemographic background. Based on the findings of this cross‐sectional study, the following conclusions were drawn:

1. From the student, patient, and clinical instructor perspectives, most of the patients' sociodemography factors did not affect the clinical communication skill domains.
2. The patients' income group played a significant role in the domain of “caring and respectful” of SCAI.

## AUTHOR CONTRIBUTIONS

Siti Mariam Ab Ghani conceptualized, designed, and coordinated the study, analyzed data, and reviewed and edited the manuscript. Puteri Nurul Adila Mohd Khairuddin coordinated the study, analyzed data, and wrote the original manuscript. Budi Aslinie Md Sabri conceptualized, designed, coordinated the study, analyzed data, and reviewed and edited the manuscript. Dieter Schonwetter conceptualized, analyzed data, and reviewed and edited the manuscript. Tong Wah Lim conceptualized, designed, coordinated the study, analyzed data, and reviewed and edited the manuscript.

## CONFLICT OF INTEREST STATEMENT

The authors declare no conflict of interest.

## ETHICS STATEMENT

This study received ethical approval (Ref. No: 600‐IRMI‐5/1/6 REC/370/16) from the Research Ethics Committee of the Universiti Teknologi MARA.

## Supporting information

Supporting information.

## Data Availability

The data that support the findings of this study are available from the corresponding author upon reasonable request.
