# Supplementary material for: Evaluating the patient sociodemographic factors affecting dental students' clinical communication skills using a three‐perspective approach
Source: Clin Exp Dent Res. 2024 Jun 16;10(3):e897. doi: 10.1002/cre2.897 (PMC11180936; doi:10.1002/cre2.897)
Supplement: Supplementary file 1 — Supporting information. [file CRE2-10-e897-s001.docx]

Appendix 1. Frequency and percentage of patient age groups.

| Age | Frequency (n) | Percentage (%) |
| --- | --- | --- |
| 18-19 | 9 | 5.1 |
| 20-29 | 36 | 20.5 |
| 30-39 | 35 | 14.2 |
| 40-49 | 40 | 22.7 |
| 50-59 | 38 | 21.6 |
| 60-69 | 25 | 14.2 |
| 70-79 | 2 | 1.1 |
| 80-99+ | 1 | 0.6 |
